# P-1480. Perception and knowledge of oncologic patients vaccination among health care workers from an oncologic hospital

**DOI:** 10.1093/ofid/ofaf695.1666

**Published:** 2026-01-11

**Authors:** Maria Jose Lopez, Melissa Reyes, Camilo Buitrago Bahamon, Maria Paula Alba, Ayda Milena Carvajal, Adriana Aya, Andrea Prada, Laura Milena Luengas

**Affiliations:** Fundacion CTIC, Bogota, Distrito Capital de Bogota, Colombia; Fundacion CTIC, Bogota, Distrito Capital de Bogota, Colombia; Fundacion CTIC, Bogota, Distrito Capital de Bogota, Colombia; Fundacion CTIC, Bogota, Distrito Capital de Bogota, Colombia; Fundacion CTIC, Bogota, Distrito Capital de Bogota, Colombia; Fundacion CTIC, Bogota, Distrito Capital de Bogota, Colombia; Fundacion CTIC, Bogota, Distrito Capital de Bogota, Colombia; Fundacion CTIC, Bogota, Distrito Capital de Bogota, Colombia

## Abstract

**Background:**

Protection against vaccine-preventable diseases is essential in susceptible patients such as oncologic ones, however there is there is a lack of knowledge and barriers to its implementation. Our objective was to assess knowledge, perception of importance, and barriers for its implementation among healthcare workers treating oncologic patients.

**Figure:**
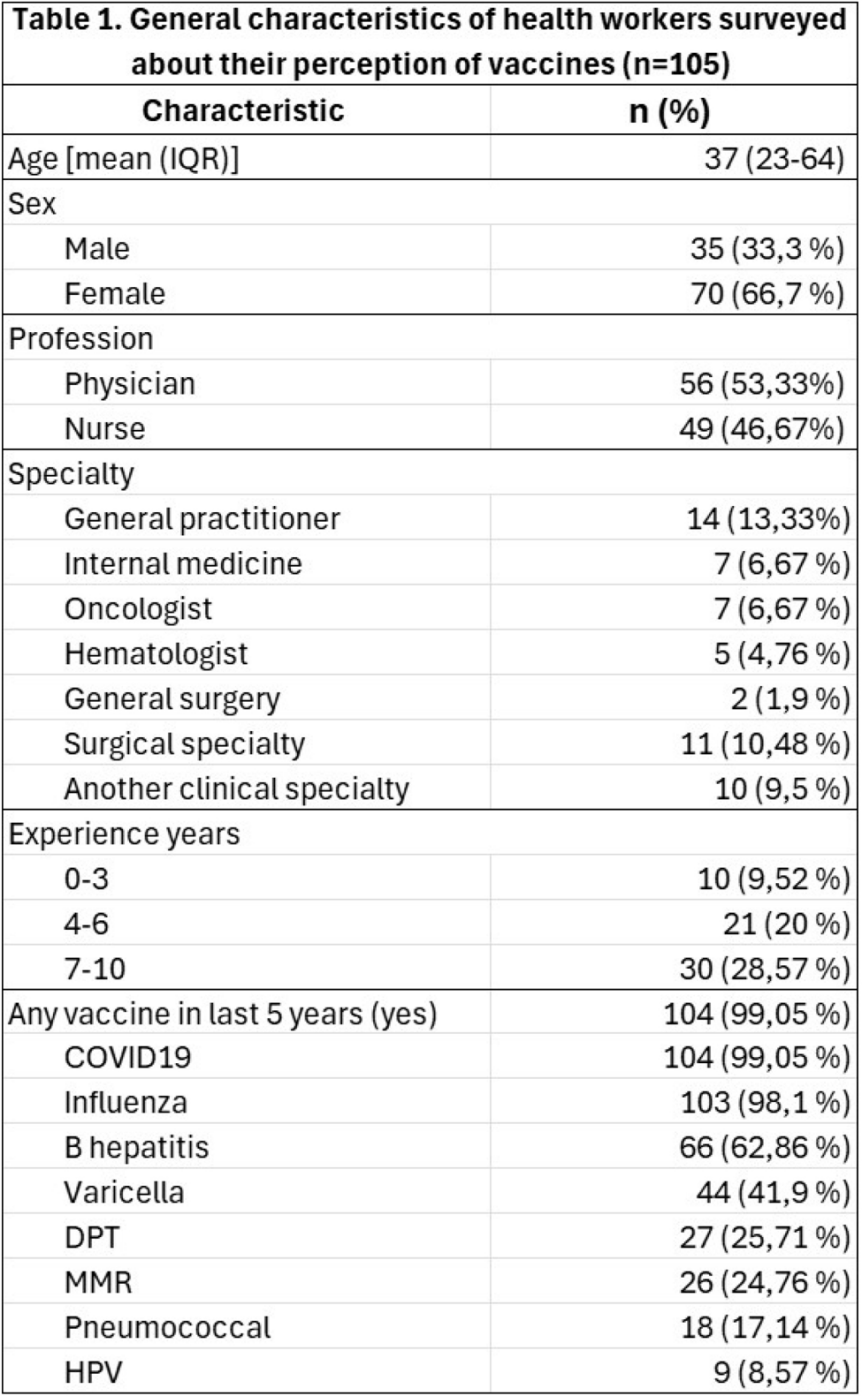
General characteristics of health workers surveyed about their perception of vaccines DPT=diphteria, pertussis, tetanus, MMR=measles, mumps, rubella, HPV= human papillomavirus, IQR=interquartile range

**Figure 1.**
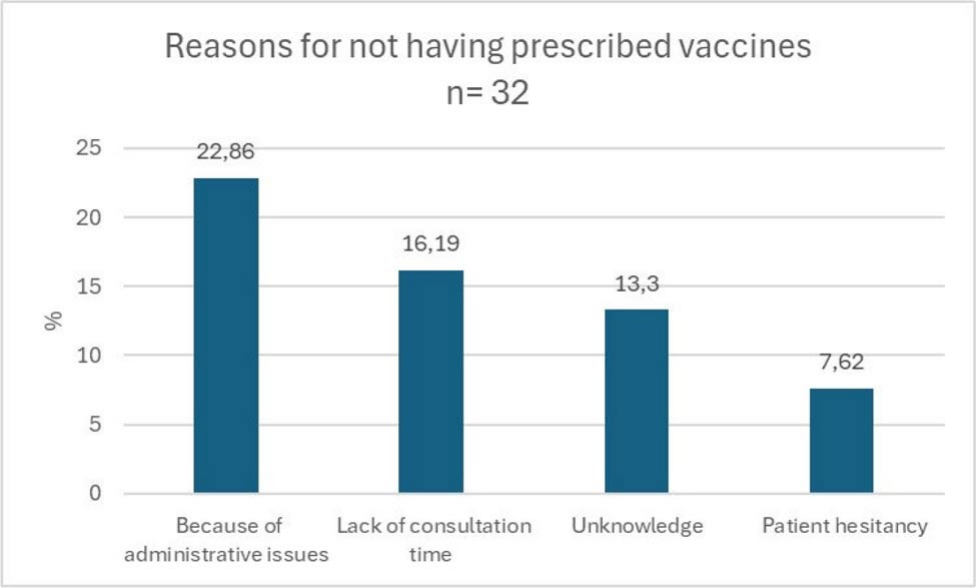
Reasons for HCW not having prescribed vaccines

**Methods:**

A cross-sectional survey was conducted among specialist physicians and nurses working in an oncology hospital in Bogota, in February 2025.

**Figure 2.**
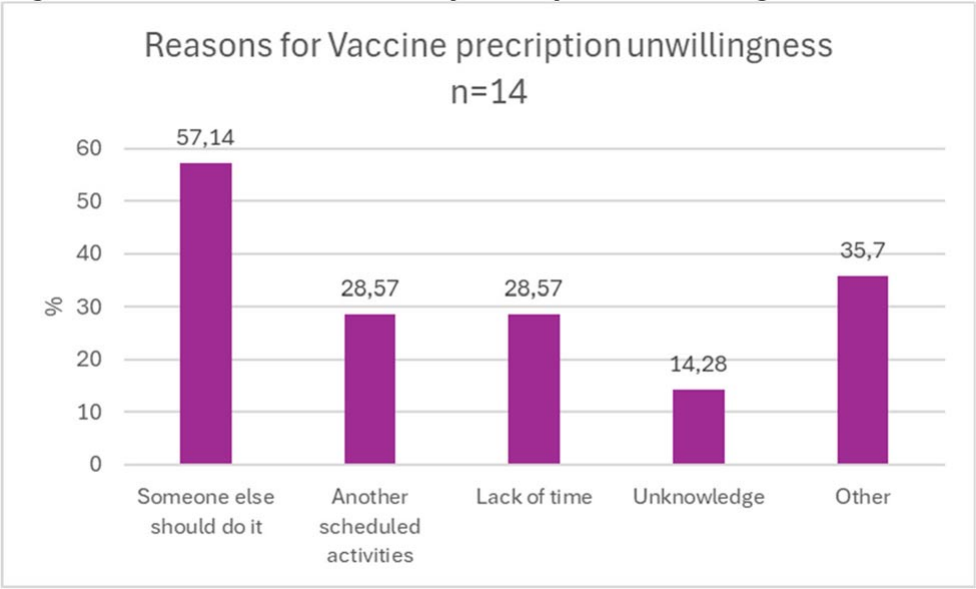
Reasons for vaccine prescription unwillingness

**Results:**

A total of 105 healthcare workers completed the survey. The respondents were 46,7 % professional nurses and 53,3 % doctors, they were mainly women (66,7 %), with postgraduate studies (71,4 %), and more of 7 years of clinical experience (70,47 %).

100 % of participants had received any vaccine in last 5 years, mainly COVID19 (99%), influenza (98%) and hepatitis B (62,9%). Among respondents 100 % thought it was important to get vaccinated and vaccinate patients, and 90,48 % reported they recommended their patients get vaccinates. However, only 22,86 % of doctors had prescribed vaccines in the last year and 75 % were willing to prescribe vaccines.

The perceived barriers for vaccine prescription were administrative issues (22,86 %), lack of consultation time (16,19 %), unknowledge (13,3%) and patient hesitancy (7,62 %). The unwillingness for vaccine prescription is because they think someone else should do it (57,14%), lack of time (28,57 %) or lack of knowledge (14,28%).

We found a mean score of 31,0/100 (IRQ 12,5 – 37,5) on the knowledge assessment, only 15,24 % knew reactogenicity concept, and 45,71 knew which vaccines were contraindicated in oncologic patients.

**Conclusion:**

Among an oncologic center healthcare workers immunization rates were high only for mandatory vaccines, there is a gap between the informed perception of importance of vaccination and the prescription and willingness to prescribe. Unknowledge is one of the reasons, and it was objectively determined, so education campaigns are required. It is also necessary to design institutional routes establishing those responsible for the prescription and compliance evaluation.

**Disclosures:**

Maria Jose Lopez, Infectious diseases service, Abbvie: Honoraria|Biomerieaux: Advisor/Consultant|Biomerieaux: Honoraria|GSK: Advisor/Consultant|GSK: Honoraria|Knight: Honoraria|Pfizer: Honoraria

